# The Tacrolimus Metabolism Rate Influences Renal Function after Kidney Transplantation

**DOI:** 10.1371/journal.pone.0111128

**Published:** 2014-10-23

**Authors:** Gerold Thölking, Christian Fortmann, Raphael Koch, Hans Ulrich Gerth, Dirk Pabst, Hermann Pavenstädt, Iyad Kabar, Anna Hüsing, Heiner Wolters, Stefan Reuter, Barbara Suwelack

**Affiliations:** 1 Department of Medicine D, Division of General Internal Medicine, Nephrology and Rheumatology, University Hospital of Münster, Münster, Germany; 2 Institute of Biostatistics and Clinical Research, University of Münster, Münster, Germany; 3 Department of Transplant Medicine, University Hospital of Münster, Münster, Germany; 4 Department of General Surgery, University Hospital of Münster, Münster, Germany; UNIFESP Federal University of São Paulo, Brazil

## Abstract

The effective calcineurin inhibitor (CNI) tacrolimus (Tac) is an integral part of the standard immunosuppressive regimen after renal transplantation (RTx). However, as a potent CNI it has nephrotoxic potential leading to impaired renal function in some cases. Therefore, it is of high clinical impact to identify factors which can predict who is endangered to develop CNI toxicity. We hypothesized that the Tac metabolism rate expressed as the blood concentration normalized by the dose (C/D ratio) is such a simple predictor. Therefore, we analyzed the impact of the C/D ratio on kidney function after RTx. Renal function was analyzed 1, 2, 3, 6, 12 and 24 months after RTx in 248 patients with an immunosuppressive regimen including basiliximab, tacrolimus, mycophenolate mofetil and prednisolone. According to keep the approach simple, patients were split into three C/D groups: fast, intermediate and slow metabolizers. Notably, compared with slow metabolizers fast metabolizers of Tac showed significantly lower estimated glomerular filtration rate (eGFR) values at all the time points analyzed. Moreover, fast metabolizers underwent more indication renal biopsies (p = 0.006) which revealed a higher incidence of CNI nephrotoxicity (p = 0.015) and BK nephropathy (p = 0.024) in this group. We herein identified the C/D ratio as an easy calculable risk factor for the development of CNI nephrotoxicity and BK nephropathy after RTx. We propose that the simple C/D ratio should be taken into account early in patient’s risk management strategies.

## Introduction

The calcineurin-inhibitor (CNI) tacrolimus (Tac) is one of the most effective immunosuppressive drugs used for renal transplantation (RTx). In combination with mycophenolate mofetil, prednisolone and daclizumab, Tac regimen showed a superior graft function, better prevention of acute rejection and superior graft survival after 12 months compared to cyclosporine A and sirolimus [1].

Unfortunately, Tac has a narrow therapeutic window. It can lead to acute as well as to chronic nephrotoxicity. Acute Tac nephrotoxicity due to high Tac levels typically presents early after RTx as e.g. isometric vacuolizations of tubular cells. In fact, even as early as one month after RTx, glomerulosclerosis in renal allografts can be associated to CNI nephrotoxicity. Typical chronic CNI-related allograft changes include tubule-interstitial fibrosis/tubular atrophy (IF/TA), tubular microcalcifications, glomerulosclerosis and artheriolar hyalinosis. Acute tubular damage can be reversed within the first three months after RTx, however, chronic lesions observed after the third month are usually progressive ones [2]–[4].

Although there seems to be a strong association between higher CNI trough levels and nephrotoxicity [5], nephrotoxic effects of Tac can occur even at prescribed low-level regimens (4–6 ng/mL) [6]. In that context it is important that Tac metabolism depends on individual factors - clinical and genetic ones. Clinical variables like haematocrit, serum albumin, age, gender, body mass index (BMI) or absorption have been proposed to be significant influencing variables [7], [8]. However, some are still a matter of debate. The most relevant genes encode the cytochrome-P450 enzymes CYP3A4 and CYP3A5 leading to significant differences in Tac pharmacodynamics [9]. However, genetic profiling of patients is still far from been a routine test and the dosage needed to reach target Tac level varies in patients with known CYP3A polymorphisms over the time [10]. Of higher clinical relevance is that physicians have to take interactions influencing Tac metabolism into account when prescribing new drugs. In the transplant setting interactions with other immunosuppressive drugs, such as corticosteroids, sirolimus and everolimus are considered in daily routine. Nevertheless, the clinical relevance of these interactions remains widely unclear [11]. Yet another unanswered question of interest is, if the presence of CYP3A5 in the apical tubular plasma membrane observed in kidneys with CNI nephrotoxicity is causally relevant or not [12], [13].

Therefore, factors which can predict patients’ risk to develop Tac side effects or promote clinically reasonable handling of Tac are of high interest to transplant surgeons and nephrologists.

We hypothesize that the Tac metabolism rate expressed as the simple blood concentration normalized by the dose (C/D ratio) is such a predictor.

## Materials and Methods

### Study population and clinical data

We analyzed data from 311 patients who underwent RTx between January 2007 and March 2012. Immunosuppressive regimen consisted of Tac (Prograf), mycophenolate mofetil (CellCept) and prednisolone (Soludecortin H/Decortin H). An induction therapy with basiliximab (Simulect) was given at day 0 and 4. Tac was started at a dose of 0.1 mg/kg bid with a target trough level of 7–12 ng/mL during the first month, 6–10 ng/mL from month 2 to 3 and 3–8 ng/mL for the following time. 1 g mycophenolate mofetil bid was administered and adapted in case of adverse events like leukopenia, diarrhea and infections. Prednisolone was started with 250 mg intravenous (i.v.) before and directly after transplantation, following 3 days of 100 mg i.v. per day. Then prednisolone was reduced by 20 mg per day. 20 mg/day was kept until the end of the first month. Afterwards, it was tapered. 10 mg qd was continued for the first 3 months and tapered to a maintenance dosage of 5 mg qd after 6 months.

Recipients and donor data were collected at the time of RTx. BMI was calculated by dividing the body weight to the square of the height. Transplant specific characterizations as HLA MM (human leukocyte antigen mismatches), patients in the European Senior Program (ESP), delayed graft function (DGF, defined as dialysis within the first week after RTx), blood type of donor and recipient, deceased or living donor transplantations, prior RTx, cold and warm ischemia times, cytomegalovirus (CMV) status before transplantation (donor and recipient), CMV infections (considered relevant ≥1.000 copies/µl), BK virus (BKV) infections (considered relevant ≥10.000 copies/µl blood) were taken from the patient's files. Panel reactive antibodies (PRA) at the time of transplantation ≥20% defined patients as antibody-positive. Histologic results were obtained from indication biopsies only. All biopsy specimens had been reviewed by one pathologist according to the revised Banff criteria [14]. Data regarding loss of transplant function, death of patients as wells as cause of death were collected. All data was collected retrospectively. Data of all patients were anonymized and de-identified prior to analysis. Our study was approved by the local ethics committee (Ethik Kommission der Ärtzekammer Westfalen-Lippe und der Medizinischen Fakultät der Westfälischen Wilhelms-Universität, No. 2014-381-f-N). Written informed consent was given by all participants at the time of transplantation for recording their clinical data and use in anonymized analysis.

Only patients receiving standard formulation of Tac (Prograf) had been included and Tac had to be part of the immunosuppressive regimen for at least 6 months. Exclusion criteria were: switch of the immunosuppressive therapy, age <16 years, and patients with an estimated glomerular filtration rate (eGFR) value lower than 10 ml/min/1.73 m^2^ or complete functional loss of the transplant were dropped out of the study. Patients were also excluded in case of lost to follow-up.

### Clinical chemistry

Whole blood was analyzed for creatinine (enzymatic assay; Creatinine-Pap, Roche Diagnostics, Mannheim, Germany) and Tac using the automated tacrolimus (TACR) assay (Dimension Clinical Chemistry System, Siemens Healthcare Diagnostic GmbH, Eschborn, Germany). Only 12-h Tac trough levels were used for analysis. Renal function was determined by eGFR calculation using the 4-variabel modification of diet in renal disease (MDRD) Study at month 1, 2, 3, 6, 12 and 24 after RTx [15].



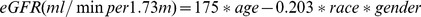


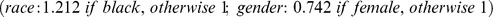



Tac metabolism rate was determined at month 1, 3 and 6 after RTx by dividing the drug blood trough concentration (C) to the corresponding daily Tac dose (D).
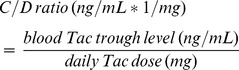



The mean C/D ratio of the three periods was used to identify three groups of Tac metabolizers (fast, intermediate and slow). In order to get reliable statistical results, groups had to encounter similar number of patients. Intermediate metabolizers were grouped around the mean C/D ratio value of 1.29 ng/mL*1/mg (1.05–1.54). A C/D ratio <1.05 ng/mL*1/mg was defined as a fast and ≥1.55 ng/mL*1/mg as a slow metabolism rate.

### Statistical analyses

Statistical analyses of retrospective and prospective data were performed using SAS software, Version 9.4 for Windows and IBM SPSS Statistics 22 for Windows (IBM Corporation, Somers, NY, USA). Inferential statistics were intended to be exploratory (to generate hypotheses) instead of confirmatory, and were interpreted accordingly. Thus, p-values were interpreted in Fisher's sense, representing the metric weight of evidence against the respective null hypothesis of no effect. Neither a global significance level nor local levels were determined. P-values were considered noticeable if <0.05 and highly noticeable if ≤0.01.

All P-values are two-sided. The primary target parameter was eGFR (ml/min/1.73 m^2^). Standard univariate statistical analyses were used to describe demographic and clinical parameters. Categorical variables are shown as absolute and relative frequencies. To quantify the evidence of differences between the three groups of Tac metabolizers (fast, intermediate and slow) Fisher’s exact tests or Chi-square tests were used. Normal-distributed continuous variables are shown as “mean ± standard deviation”. Not normal-distributed continuous variables are reported as “median [minimum – maximum]”. The eGFR values were compared at each time point (1 month, 2, 3, 6, 12, 24 months) between the three Tac groups. Due to their distributional properties one-way ANOVAs (analysis of variances) or Kruskal-Wallis test were applied. Further, the corresponding pairwise comparisons were performed using T-tests or Mann-Whitney U tests without adjusting for multiple testing.

Additionally, multivariable analyses were carried out for longitudinal data. The aim was to identify predictor variables as well as possibly confounders on the eGFR. To account for missing values (treated as missing at random) and repeated outcome values, linear mixed models were fitted. The dependent variable was eGFR (ml/min/1.73 m^2^). Variable selections were performed to detect independent variables with the strongest impact on eGFR. Potential independent variables were: age at RTx (years), gender of the donor (female, male) weight (kg), height (cm), living donor (yes, no), cold ischemia time (hours), warm ischemia time (min), time points (month 1, 2, 3, 6, 12 and 24 after transplantation), the Tac metabolism group (fast, intermediate and slow) and the interaction effect (Tac metabolism group*time point). The final linear mixed model includes the main effects age, weight, living donor transplantation, time point, Tac metabolism group and the interaction term (Tac metabolism group*time point). The repeated measurements were modeled using a compound symmetry covariance structure (CS) with patient as subject and the time point as repeated variable.

## Results

### Study group

Between January 2007 and March 2012, 406 patients underwent a renal transplantation. 311 of these patients received an initial immunosuppressive regimen with Tac, mycophenolate mofetil, prednisolone and basiliximab. 16 patients did not reach the end of the first month. One of these patients died after 4 days due to a cerebral insult, 7 patients were switched to another immunosuppression and 8 patients showed a loss of kidney function.

248 patients fulfilled the study criteria by remaining on Tac for at least 6 months after RTx. The patients included were between 16 and 78 years of age (51.9±14.2) and 148 (59.7%) were male. The eGFR values were collected of 295 patients after the first and second month, of 286 patients after month 3, 248 patients after month 6, 229 patients after month 12 and of 161 patients 24 months after RTx.

The only relevant drugs interacting with the Tac metabolism were corticosteroids which were used in all three groups.

### Tacrolimus metabolism rate

**Figure 1:**
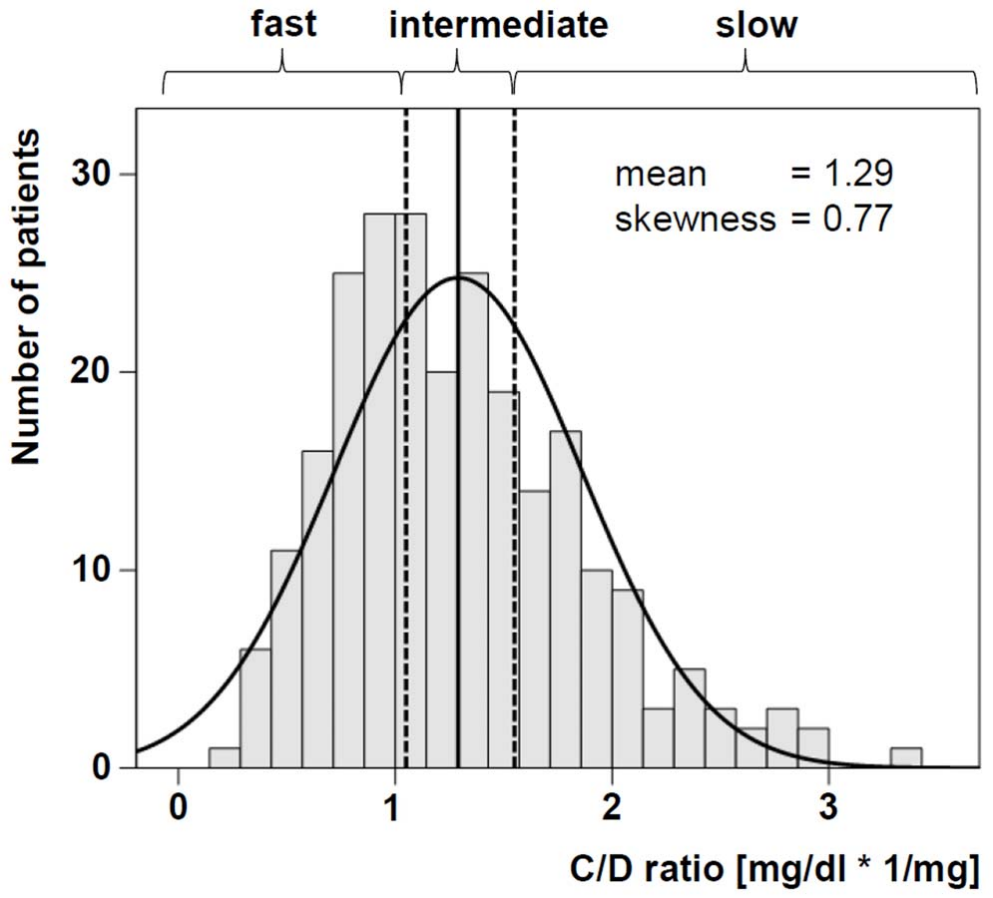
Histogram of the distribution of the Tac C/D ratio (ng/mL*1/mg). The patients showed a symmetric distribution relating to their C/D ratio and were categorized in three groups: slow, intermediate and fast metabolizers. Intermediate metabolizers were grouped around the mean C/D ratio value (1.05–1.54). Patients showing a C/D ratio <1.05 were defined as fast metabolizers, ≥1.55 as slow metabolizers.

The C/D ratio was used to define patients with different Tac metabolism rates. A C/D ratio minimum of 0.25 and a maximum of 3.37 ng/mL*1/mg (mean value 1.29 ng/mL*1/mg) was identified. The 248 included patients were categorized in three groups: fast (n = 97), intermediate (n = 78) and slow metabolizers (n = 73). The C/D ratio values showed a symmetric distribution (Figure 1).

### Descriptive statistics

Table 1 shows the characteristics of the 248 patients who remained on Tac for ≥6 months after RTx. Univariate analyses revealed a statistically noticeable correlation of the recipient's age and the metabolism rate. Slow metabolizers were older than fast (p = 0.006) and intermediate metabolizers (p<0.001). Furthermore, in a paired comparison the cohort of slow metabolizers included fewer living donor transplantations than the fast (p = 0.015) and intermediate metabolizers (p = 0.002).

**Table 1:** Patients characteristics.

	fast metabolizers	interm. metabolizers	slow metabolizers	P-value
	(n = 97)	(n = 78)	(n = 73)	
weight (kg)	74.4±15.7	76.4±15.1	74.0±14.8	0.562^a^
height (m)	1.74±0.1	1.74±0.1	1.70±0.1	0.062^a^
BMI (kg/m^2^)	24.6±4.3	25.1±4.1	25.4±4.4	0.451^a^
age (years)	50.7±14.1	48.2±15.3	57.5±11.3	<0.001^a^
gender (m/f)	54 (56%)/43 (44%)	49 (63%)/29 (37%)	45 (62%)/28 (38%)	0.581^c^
living donor transpl.	25 (26%)	25 (32%)	8 (11%)	0.007^c^
ESP transplantations	18 (19%)	12 (15%)	19 (26%)	0.242^c^
AB0-incompatible transplantations	1	2	1	0.712^c^
cold ischemic time (h)	8.3±5.4	7.9±5.5	9.6±4.9	0.074^a^
warm ischemic time (min)	30.0 (14–50)	30.0 (18–75)	30.0 (17–60)	0.378^b^
number of transplatations				
one	82	67	56	
two	11	10	16	0.251^c^
three	4	1	1	
donor characteristics				
donor age (years)	54.0±13.2	51.0±17.0	54.1±18.2	0.389^a^
donor gender (m/f)	39 (40%)/58 (60%)	39 (50%)/39 (50%)	33 (45%)/40 (55%)	0.431^c^

BMI, body mass index; h, hours; min, minutes; ESP, European Senior Programm; ^a^ P-value is from the one-way ANOVA; ^b^ P-value is from the Kruskal-Wallis test; ^c^ P-value is from the Fisher's Exact test.

### Doses and blood concentrations

The average maintaining dose for Tac was higher in the group of fast metabolizers after 1, 3 and 6 months than in the other two groups (p<0.001, Table 2). The mean daily dose of fast metabolizers was approximately twice as high as the dose of slow metabolizers.

**Table 2:** Medication doses and blood trough concentrations.

	fast metabolizers	interm. metabolizers	slow metabolizers	P-value
	(n = 97)	(n = 78)	(n = 73)	
tacrolimus mean trough level (ng/ml)	8.2±1.6	9.2±1.8	9.5±1.8	<0.001^a^
after 1 month	9.4±3.2	10.5±2.7	11.0±3.2	0.002^a^
after 3 months	7.8±2.1	9.1±2.9	9.5±2.8	<0.001^a^
after 6 months	7.2±2.3	7.8±2.4	8.0±2.8	0.079^a^
tacrolimus mean daily dose (mg)	11.0 (6.3–26.7)	7.5 (4–14)	5.5 (2.33–11.5)	<0.001^b^
after 1 month	14 (6–40)	10 (4–22)	8 (2–20)	<0.001^b^
after 3 months	10 (3.5–23)	7 (3.5–13)	4.25 (2–12)	<0.001^b^
after 6 months	9 (3–21)	5 (2–9.5)	3 (1.5–7.5)	<0.001^b^
prednisolon mean daily dose (mg)	15 (3.75–36.7)	14.17 (5–70)	13.33 (0–40)	0.06^b^
after 1 month	20 (15–90)	20 (15–70)	20 (0–50)	0.155^b^
after 3 months	13.75 (2.5–30)	12.5 (5–30)	12.5 (0–30)	0.496^b^
after 6 months	10 (5–30)	8.5 (5–20)	7.5 (0–20)	0.114^b^

a P-value is from the one-way ANOVA. ^b^ P-value is from the Kruskal-Wallis test.

The Tac trough levels were analyzed at the same time points. Statistically noticeable differences were only found 1 month and 3 months after RTx, when the mean prednisolone dose was more than 20 and 10 mg/day, respectively (Table 2). The Tac target trough levels were met at all time points. There were no differences of applied prednisolone doses between the three groups.

### Renal function

**Figure 2:**
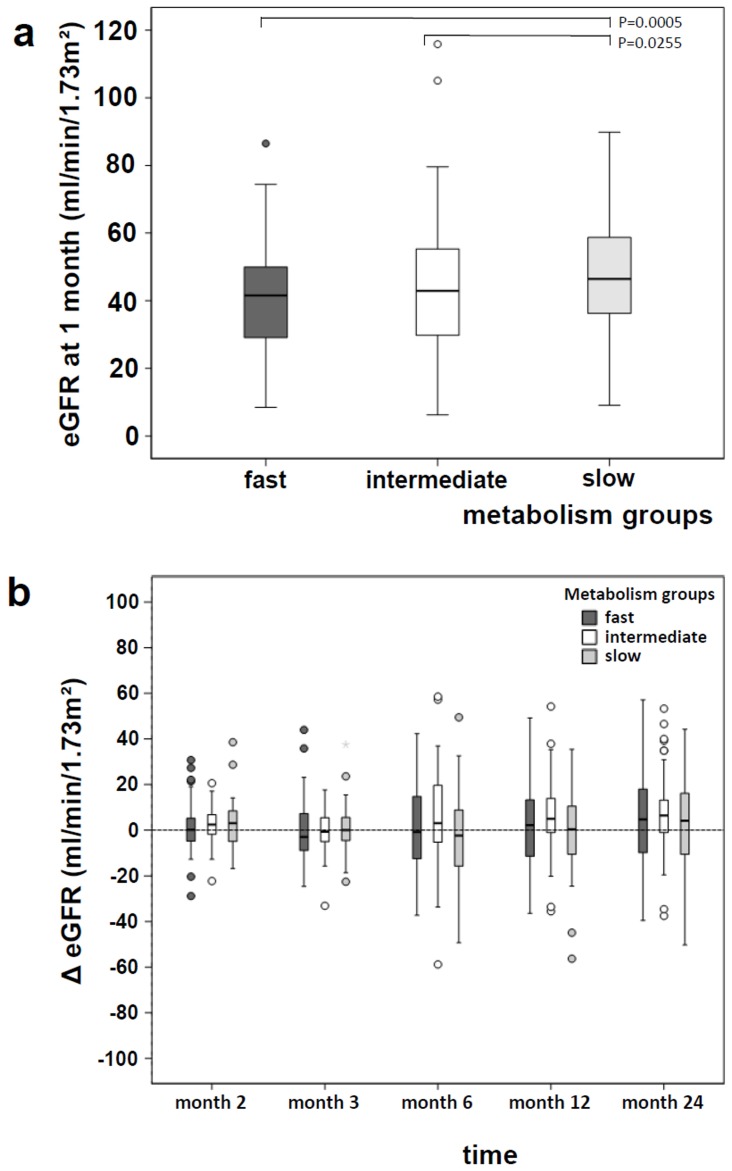
eGFR value comparison between the three metabolism groups (a). Fast and intermediate Tac metabolizers showed statistically noticeable lower eGFR values compared to slow metabolizers one month after RTx. P-values refer to the linear mixed model. Renal function in a 24 months follow-up (b). The differences between the follow-up eGFR values (month 2, 3, 6, 12, 24) and the eGFR value and the eGFR value at month 1 are shown for every metabolism group.

The eGFR values showed a symmetric distribution, so the assumption of an approximate normal distribution could be justified. Consequently, a linear mixed model was fitted to adjust for possible imbalances between the independent variables that may have an influence on the renal function and to identify possible predictor variables. In the final model the main effects of metabolism group (p<0.001) and of time point (p<0.001), as well as the interaction (p = 0.054) showed an influence on the renal function (Table 3, linear mixed model). The model based estimates of the pairwise differences of eGFR between the metabolism groups were calculated for each time point. At one month after RTx fast and intermediate metabolizers already revealed a statistically noticeable decreased renal function compared to slow metabolizers (Figure 2a). In the 24 months follow-up fast metabolizers had statistically noticeable lower eGFR values (Table 3, linear mixed model).

**Table 3:** Renal function (linear mixed model).

	Model-based estimates			
	mean eGFR	lower 95% confidence limit	upper 95% confidence limit	P-value
living donors (yes vs. no)	3.7	−0.3	7.6	0.068
weight (per kg)	−0.3	−0.3	−0.1	<0.0001
age at TX (per year)	−0.3	−0.4	−0.2	<0.0001
tacrolimus metabolism group				<0.0001
time points				<0.0001
tacrolimus metabolism groups*time points				0.054
Combinded estimates of main effects and interaction term of tacrolimus metabolism group and time points				
at 1 month				
intermediate vs. slow	−6.2	−11.6	−0.8	0.026
fast vs. slow	−9.0	−14.1	−3.9	0.0005
fast vs. intermediate	−2.9	−7.8	2.1	0.26
at 2 months				
intermediate vs. slow	−6.9	−12.4	−1.4	0.014
fast vs. slow	−11.1	−16.3	−6.0	<0.0001
fast vs. intermediate	−4.3	−9.3	0.7	0.096
at 3 months				
intermediate vs. slow	−6.9	−12.5	−1.3	0.015
fast vs. slow	−10.9	−16.1	−5.7	<0.0001
fast vs. intermediate	−4.0	−9.1	1.0	0.118
at 6 months				
intermediate vs. slow	1.2	−4.2	6.6	0.656
fast vs. slow	−9.0	−14.1	−3.9	0.0005
fast vs. intermediate	−10.2	−15.2	−5.3	<0.0001
at 12 months				
intermediate vs. slow	−0.5	−6.0	5.0	0.861
fast vs. slow	−7.6	−12.8	−2.4	0.0043
fast vs. intermediate	−7.1	−12.1	−2.1	0.0053
at 24 months				
intermediate vs. slow	−1.8	−8.2	4.5	0.566
fast vs. slow	−8.8	−14.7	−2.8	0.0039
fast vs. intermediate	−6.9	−12.5	−1.3	0.016

Results of the final linear mixed model after variable selection. The parameter estimates for fixed effects of eGFR (ml/min/1.73 m^2^) are shown. P-values are from the Wald tests. Repeated measurements were modeled using a compound symmetry covariance structure (CS) with patient as subject and the time point as repeated variable.

Interestingly, the mean C/D ratio of the 25 patients with the worst renal outcome (who developed an eGFR value of less than 30 ml/min/1.73 m^2^ or even turned back to dialysis) was 1.10±0.46 and of patients with biopsy proven CNI nephrotoxicity 0.92±0.48 ng/mL*1/mg.

Possibly, due to a drop out of patients who switched their immunosuppression or died, the eGFR values of all three groups showed a highly noticeable increase in the 24 month follow-up. The univariate changes of the eGFR values since month 1 are shown in Figure 2b. In the linear mixed model also the main effects age (p<0.001), weight (p<0.001) and living donor transplantation (p = 0.067) had a potential influence on the renal function. Despite the small p-value the effect of age was very small. Per year of age the eGFR value was 0.3 mL/min/1.73 m^2^ lower. The nominal effect of weight was also quite small. Per kg weight the eGFR value was 0.3 mL/min/1.73 m^2^ smaller. The influence of living donor transplantation indicated only a trend. In these patients the eGFR was 3.7 mL/min/1.73 m^2^ per year higher than in patients who received a deceased donor transplant.

### Post-transplantation clinical characteristics

There were no statistically noticeable differences between the three groups concerning the number of PRA, HLA MM, DGF or CMV infections (Table 4). In the group of fast metabolizers noticeable more patients underwent an indication renal biopsy (p = 0.006). Although there were no differences between histologically proven T cell-mediated rejection or antibody-mediated rejection, in the group of fast metabolizers more cases of CNI nephrotoxicity (p = 0.015) and BK nephropathy (p = 0.024) occured.

**Table 4:** Antibodies, HLA MM, DGF, infections and biopsy results.

	fast metabolizers	interm. metabolizers	slow metabolizers	P-value
	(n = 122)	(n = 91)	(n = 82)	
PRA (>20%)	2 (2%)	3 (3%)	2 (2%)	–
HLA MM				
no HLA MM	15 (12%)	13 (14%)	10 (12%)	
1–3 HLA MM	67 (55%)	45 (50%)	50 (61%)	0.651
4–6 HLA MM	40 (33%)	33 (36%)	22 (27%)	
DGF	25 (21%)	15 (17%)	11 (13%)	0.434
CMV				
CMV high risk	30 (25%)	16 (18%)	20 (24%)	
CMV interm. risk	77 (63%)	61 (69%)	51 (62%)	0.784
CMV low risk	15 (12%)	12 (13%)	11 (13%)	
CMV infection	15 (12%)	11 (12%)	5 (6%)	0.292
BK viremia	8 (7%)	7 (8%)	1 (1%)	0.11
biopsy results				
indication biopsies	53 (43%)	30 (33%)	18 (22%)	0.006
TMR	2 (2%)	4 (4%)	1 (1%)	–
AMR	5 (4%)	3 (3%)	0	–
CNI-nephrotoxicity	13 (11%)	2 (2%)	2 (2%)	0.015
BK nephropathy	5 (4%)	0	0	0.024
others	28 (23%)	21 (23%)	15 (18%)	–

PRA, panel reactive antibodies; HLA MM, human leucocyte antigen mismatch; DGF, delayed graft function; CMV, cytomegalovirus; TMR, T cell-mediated rejection; AMR, antibody-mediated rejection; IF/TA, interstitial fibrosis/tubular atrophy; P-values are from the Fisher's Exact tests; due to small frequencies some P-values are not reported.

From time point 1 month until month 24, 58 of 295 patients (19.7%) were switched from Tac to another immunosuppressive drug (sirolimus, everolimus or cyclosporin A). The main reason for a switch was CNI nephrotoxicity (assumed or identified in kidney biopsies). In particular, in fast metabolizers the immunosuppressive regimen was significantly more often changed than in slow metabolizers (p = 0.03; Table 5).

**Table 5:** Reasons for switching immunosuppression and adverse events.

	fast metabolizers	interm. metabolizers	slow metabolizers	P-value
	(n = 122)	(n = 91)	(n = 82)	
switch of IS	28 (23%)	16 (18%)	14 (17%)	0.523
assumed and proven CNI-tox	11 (9%)	4 (4%)	1 (1%)	0.047
adverse events	8 (7%)	5 (6%)	4 (5%)	0.948
acute rejections	1 (1%)	0	0	–
chronic allograft failure	2 (2%)	1 (1%)	0	–
infections	5 (4%)	2 (2%)	5 (5%)	–
maligne diseases	0	3 (3.3%)	1 (1.2%)	–
compliance	1 (1%)	1 (1%)	3 (4%)	–
loss of funcion	6 (5%)	2 (2%)	3 (4%)	0.636
chronic allograft failure	2 (2%)	0	0	–
acute rejections	2 (2%)	1 (1%)	2 (2%)	–
primary non-function	1 (1%)	1 (1%)	1 (1%)	–
CNI nephrotoxicity	1 (1%)	0	0	–
death	9 (7%)	4 (4%)	4 (5%)	0.665
infections	6 (5%)	2 (2%)	0	0.111
maligne diseases	1 (1%)	0	1 (1%)	–
bleeding	0	1 (1%)	1 (1%)	–
cardiovascular	2 (2%)	1 (1%)	2 (2%)	–

CNI, calcineurin inhibitor; IS, immunosuppression; P-values are from the Fisher's Exact test; due to small frequencies some P-values are not reported.

Eleven patients (3.7%) lost their renal function within 2 years after RTx. Of these, more fast metabolizers returned to dialysis treatment (trend). Moreover, chronic allograft failure and CNI nephrotoxicity appeared only in the group of fast metabolizers to be reasons for a loss of renal function.

Mortality rate was highest in the group of fast metabolizers when compared between the groups (7.4% vs. 4.4% vs. 4.9%). The leading cause of death was infection (53.3%), followed by cardiovascular events (33.3%), with no statistical differences between the groups (Table 5).

## Discussion

Our data indicate that Tac metabolism ratio of recipients of renal allografts influences kidney function. We identified a C/D ratio <1.05 corresponding to fast metabolizers of Tac to be associated with a decreased renal function in a 24 month follow-up after RTx when compared to higher C/D ratios. We conclude from our data that the C/D ratio in patients receiving Tac is a simple and inexpensive tool to identify patients at risk for the development of CNI nephrotoxicity or BK nephropathy. Moreover, a C/D ratio <1.05 was associated with lower renal function and higher mortality in a 24 months follow-up in our cohort when compared to patients with higher C/D ≥1.05.

Former studies revealed several clinical factors which impact on the individual Tac metabolism rate. Patients identified as fast metabolizers encountered noticeable more females and had predominantly an age <60 years and a BMI <25 kg/m^2^. The corticosteroid dose is also known to have an influence on the Tac metabolism and is the only clinical factor which might be changed rapidly in patients with stable immunosuppressive regimen [16]. Besides, other drugs can interfere with Tac metabolism. Therefore, it is essential to check new drugs introduced in these patients for Tac interactions (i.e. CYP3A inhibitors or inductors) and perform Tac level monitoring in the follow-up. Independently from predictable factors which can change the Tac metabolism rate, the following ones have a relatively constant effect over the time. Age for example. Comparing the three Tac metabolizer groups in our study, younger patients indicated to have a faster metabolism rate than older ones. By contrast, gender had no effect in our cohort. This is according to observations from Gijsen et al. who identified in pediatric heart recipients age and not sex to be related to Tac dosing [8]. Interestingly, the authors could not find any association between renal function and Tac trough level as well as the genotype of CYP3A expressed. Similar results had been published for RTx patients. Mourad et al. observed a correlation between CYP3A genotype and Tac trough concentration (as well as age and weight did) but failed to demonstrate an impact of Tac level to renal function within 14 days after RTx. However, the short follow-up and the presumably changing corticosteroid doses in this time period, which influence Tac metabolism, are limitations of this study [17]. Interestingly, even a 5-year follow-up in RTx patient failed to show a correlation between CYP3A genotype and serum creatinine limiting the clinical relevance of this information when standard therapeutic drug monitoring is applied [10]. However, it is out of question that genetic factors impact on the Tac metabolism. In this regard the relevance of the fact that fast metabolizers predominantly, but not exclusively, express the genotype CYP3A5*1 remains unclear [18].

Therefore, we think that genetic testing of patients does not add any relevant data to our approach and counteracts our simplification strategy. In this context, patient's weight was excluded from the formula, as opposed to previous studies [7], [16]. However, our approach is supported by recently published data about pharmacokinetics of different intestinal and hepatic CYP3A5 genotypes who provide evidence that body weight can be removed from the C/D ratio formula [19]. This is supported by data from Kim et al. who could not demonstrate a relation between weight and Tac side effects in a 5-year follow-up of RTx patients [10]. In our study, there was no statistically noticeable difference between the factors weight and BMI between the three groups, which allowed us to consider these as non-mandatory factors for inclusion in the formula.

In our opinion, identification of fast metabolizers by simply using the C/D ratio is relevant. Fast metabolizers with a C/D ratio <1.05 showed lower eGFR values from the first until the 24^th^ month after RTx than intermediate or slow metabolizers. Patients of this group underwent frequently more indication renal biopsies which revealed more often cases of CNI nephrotoxicity despite lower Tac levels (Table 2). In fact, the mean C/D ratio of patients with biopsy proven CNI nephrotoxicity in our study was <1.05. It would have been interesting to know, if on the other hand a C/D ratio of <1.05 protects from acute rejection. However, due to the small number of acute rejections we cannot sufficiently comment on a relation between C/D ratio and rejection. As expected patients with a C/D ratio <1.05 needed higher daily Tac doses than the patients of the other two groups. The ELITE-Symphony study supports our data by showing lower GFR values in the group of patients receiving a higher CNI dose [1]. Kuypers et al. identified an association between a high daily Tac dosage and histologically proven CNI nephrotoxicity [18]. Taking the available data into account, high daily Tac doses (in relation to the serum level) applied in fast metabolizers may account for lower renal function after RTx.

Furthermore, histologically proven BK nephropathy more often occurred in fast metabolizers than in the intermediate or slow metabolizers. Immunosuppressive regimens including Tac and MMF are associated with an increased risk of reactivation of BKV and BK nephropathy [20]. Borni-Duval et al. identified Tac trough levels of more than 10 ng/mL as a risk factor for BK nephropathy [21]. Taking together, it remains of interest and should be addressed in further studies, if the C/D ratio could be a more accurate predictor of BK nephropathy or not. The fact that CNI nephropathy and BK nephropathy as well as a higher incidence of death due to infection is related to lower C/D ratios led us to the hypothesis that fast metabolizers might suffer from overexposure and related over immunosuppression or toxicity at least at some time of the day. This should be addressed in further studies. It was questioned if Tac absorption might influence the Tac metabolism. Enteric Tac absorption has been characterized and factors like gastric emptying rate have been investigated. These processes can influence early peak level but seem not to affect trough level to a relevant extent [22]–[24].

During the follow-up of 24 months after transplantation a slightly increasing mean-eGFR value was calculated in all three groups. The most likely explanation is the drop-out of patients due to a switch of the immunosuppression or death.

Compared to other studies, there was only a low rate of serious events, such as loss of renal function (3.4%) and death (5.8%) in a follow-up of 24 months [25]. By trend, in the group of fast metabolizers, more patients died of infections than of other reasons.

The mainly retrospective analysis of this study leads to a few limitations. The study population was Caucasian. Therefore, the study data is only reliable for this ethnical group with similar genetic characteristics. According to the standard procedure of our hospital this study contains indication renal biopsies only. Thus, graft changes which have not been translated into significant eGFR decreases yet could have been missed and due to the small rejection rate, we cannot comment on the influence of C/D ratio on rejection.To sum up, we demonstrated that Tac metabolism rate defined by C/D ratio influences renal function within a 24 months follow-up after RTx whereas fast metabolism was associated with a lower eGFR. If the discrimination between intermediate and slow metabolizers will impact on therapy and outcome should be evaluated in the future. Furthermore, we generated hypotheses and randomized, multicenter studies should follow to confirm these results and answer the question among others if fast Tac metabolizers show a greater profit from a switch to a mammalian target of rapamycin inhibitor than intermediate or slow metabolizers.

We conclude that in order to achieve long term survival of patients and their renal transplant it is important to minimize risk factors potentially affecting graft survival and patient’s safety. One central factor to succeed is an tailored immunosuppressive regimen taking the patient’s individual situation into account. However, in our experience only simple and cost effective approaches have a real chance to be adopted for daily routine by clinicians. If the proposed C/D ratio is able to fulfil these criteria and needs, it should to be evaluated in further prospective studies.

## References

[1] Ekberg H, Tedesco-Silva H, Demirbas A, Vitko S, Nashan B, et al (2007) Reduced exposure to calcineurin inhibitors in renal transplantation. N Engl J Med 357: 2562–2575. 18094377 10.1056/NEJMoa067411

[2] Naesens M, Kambham N, Concepcion W, Salvatierra O Jr, Sarwal M (2007) The evolution of nonimmune histological injury and its clinical relevance in adult-sized kidney grafts in pediatric recipients. Am J Transplant 7: 2504–2514. 17725681 10.1111/j.1600-6143.2007.01949.x

[3] Nankivell BJ, Borrows RJ, Fung CL, O'Connell PJ, Allen RD, et al (2003) The natural history of chronic allograft nephropathy. N Engl J Med 349: 2326–2333. 14668458 10.1056/NEJMoa020009

[4] Nankivell BJ, Borrows RJ, Fung CL, O'Connell PJ, Allen RD, et al (2004) Evolution and pathophysiology of renal-transplant glomerulosclerosis. Transplantation 78: 461–468. 15316377 10.1097/01.tp.0000128612.75163.26

[5] Jacobson PA, Schladt D, Israni A, Oetting WS, Lin YC, et al (2012) Genetic and clinical determinants of early, acute calcineurin inhibitor-related nephrotoxicity: results from a kidney transplant consortium. Transplantation 93: 624–631. 22334041 10.1097/TP.0b013e3182461288 PMC3299910

[6] Tsuchiya T, Ishida H, Tanabe T, Shimizu T, Honda K, et al (2013) Comparison of pharmacokinetics and pathology for low-dose tacrolimus once-daily and twice-daily in living kidney transplantation: prospective trial in once-daily versus twice-daily tacrolimus. Transplantation 96: 198–204. 23792649 10.1097/TP.0b013e318296c9d5

[7] Stratta P, Quaglia M, Cena T, Antoniotti R, Fenoglio R, et al (2012) The interactions of age, sex, body mass index, genetics, and steroid weight-based doses on tacrolimus dosing requirement after adult kidney transplantation. Eur J Clin Pharmacol 68: 671–680. 22101623 10.1007/s00228-011-1150-0

[8] Gijsen V, Mital S, van Schaik RH, Soldin OP, Soldin SJ, et al (2011) Age and CYP3A5 genotype affect tacrolimus dosing requirements after transplant in pediatric heart recipients. J Heart Lung Transplant 30: 1352–1359. 21930396 10.1016/j.healun.2011.08.001 PMC3640375

[9] Tavira B, Coto E, Diaz-Corte C, Ortega F, Arias M, et al (2011) Pharmacogenetics of tacrolimus after renal transplantation: analysis of polymorphisms in genes encoding 16 drug metabolizing enzymes. Clin Chem Lab Med 49: 825–833. 21480817 10.1515/CCLM.2011.143

[10] Kim IW, Noh H, Ji E, Han N, Hong SH, et al (2012) Identification of factors affecting tacrolimus level and 5-year clinical outcome in kidney transplant patients. Basic Clin Pharmacol Toxicol 111: 217–223. 22469198 10.1111/j.1742-7843.2012.00892.x

[11] Kuypers DR (2008) Influence of interactions between immunosuppressive drugs on therapeutic drug monitoring. Ann Transplant 13: 11–18. 18806728

[12] Metalidis C, Lerut E, Naesens M, Kuypers DR (2011) Expression of CYP3A5 and P-glycoprotein in renal allografts with histological signs of calcineurin inhibitor nephrotoxicity. Transplantation 91: 1098–1102. 21544031 10.1097/TP.0b013e3182177502

[13] Joy MS, Hogan SL, Thompson BD, Finn WF, Nickeleit V (2007) Cytochrome P450 3A5 expression in the kidneys of patients with calcineurin inhibitor nephrotoxicity. Nephrol Dial Transplant 22: 1963–1968. 17395652 10.1093/ndt/gfm133

[14] Sis B, Mengel M, Haas M, Colvin RB, Halloran PF, et al (2010) Banff '09 meeting report: antibody mediated graft deterioration and implementation of Banff working groups. Am J Transplant 10: 464–471. 20121738 10.1111/j.1600-6143.2009.02987.x

[15] Levey AS, Coresh J, Greene T, Stevens LA, Zhang YL, et al (2006) Using standardized serum creatinine values in the modification of diet in renal disease study equation for estimating glomerular filtration rate. Ann Intern Med 145: 247–254. 16908915 10.7326/0003-4819-145-4-200608150-00004

[16] Anglicheau D, Flamant M, Schlageter MH, Martinez F, Cassinat B, et al (2003) Pharmacokinetic interaction between corticosteroids and tacrolimus after renal transplantation. Nephrol Dial Transplant 18: 2409–2414. 14551375 10.1093/ndt/gfg381

[17] Mourad M, Wallemacq P, De Meyer M, Brandt D, Van Kerkhove V, et al (2006) The influence of genetic polymorphisms of cytochrome P450 3A5 and ABCB1 on starting dose- and weight-standardized tacrolimus trough concentrations after kidney transplantation in relation to renal function. Clin Chem Lab Med 44: 1192–1198. 17032130 10.1515/CCLM.2006.229

[18] Kuypers DR, de Jonge H, Naesens M, Lerut E, Verbeke K, et al (2007) CYP3A5 and CYP3A4 but not MDR1 single-nucleotide polymorphisms determine long-term tacrolimus disposition and drug-related nephrotoxicity in renal recipients. Clin Pharmacol Ther 82: 711–725. 17495880 10.1038/sj.clpt.6100216

[19] Ji E, Choi L, Suh KS, Cho JY, Han N, et al (2012) Combinational effect of intestinal and hepatic CYP3A5 genotypes on tacrolimus pharmacokinetics in recipients of living donor liver transplantation. Transplantation 94: 866–872. 22992768 10.1097/TP.0b013e318263700a PMC3478502

[20] Acott P, Babel N (2012) BK virus replication following kidney transplant: does the choice of immunosuppressive regimen influence outcomes? Ann Transplant 17: 86–99. 10.12659/aot.882640 22466913

[21] Borni-Duval C, Caillard S, Olagne J, Perrin P, Braun-Parvez L, et al (2013) Risk factors for BK virus infection in the era of therapeutic drug monitoring. Transplantation 95: 1498–1505. 23778568 10.1097/TP.0b013e3182921995

[22] Watanabe Y, Sato M, Abe Y, Yamamoto T, Kashu Y, et al (1998) Enteric absorption of FK 506: estimation by a block liver perfusion technique in rats. Transplant Proc 30: 3777–3778. 9838656 10.1016/s0041-1345(98)01233-0

[23] Karamperis N, Povlsen JV, Hojskov C, Poulsen JH, Pedersen AR, et al (2003) Comparison of the pharmacokinetics of tacrolimus and cyclosporine at equivalent molecular doses. Transplant Proc 35: 1314–1318. 12826146 10.1016/s0041-1345(03)00481-0

[24] Kuypers DR, Claes K, Evenepoel P, Maes B, Vanrenterghem Y (2004) The rate of gastric emptying determines the timing but not the extent of oral tacrolimus absorption: simultaneous measurement of drug exposure and gastric emptying by carbon-14-octanoic acid breath test in stable renal allograft recipients. Drug Metab Dispos 32: 1421–1425. 15383495 10.1124/dmd.104.001503

[25] Ekberg H, Bernasconi C, Tedesco-Silva H, Vitko S, Hugo C, et al (2009) Calcineurin inhibitor minimization in the Symphony study: observational results 3 years after transplantation. Am J Transplant 9: 1876–1885. 19563339 10.1111/j.1600-6143.2009.02726.x

